# Comments on the tolerance of Clostridioides difficile spores to disinfection with sodium hypochlorite disinfectant

**DOI:** 10.1099/mic.0.001435

**Published:** 2024-05-21

**Authors:** J.W. Decousser, F. Barbut, R. Baron, P. Parneix, T. Lavigne, S. Romano-Bertrand, for the Scientific Committee of the French Society for Hospital Hygiene and the National Reference Laboratory for Clostridioides difficile

**Affiliations:** 1 Équipe Opérationnelle d’Hygiène, Hôpitaux Universitaires Henri-Mondor, Assistance Publique-Hôpitaux de Paris, UR DYNAMYC 7380, Faculté de Santé, Univ Paris-Est Créteil (UPEC), Enva, USC ANSES, Créteil, France; 2 National Reference Laboratory for Clostridioides difficile, Assistance Publique-Hôpitaux de Paris, Hôpital Saint-Antoine, 75012 Paris, France; Université Paris Cité, INSERM UMR-1139, 75006 Paris, France; 3 Service Hygiène Hospitalière, Pôle Recherche et Santé Publique, Centre Hospitalier Universitaire de Brest, Brest, France; 4 Nouvelle Aquitaine Healthcare-Associated Infection Control Centre, Bordeaux University Hospital, Bordeaux, France; 5 Service d’Hygiène Hospitalière, Hôpitaux Universitaires de Strasbourg, Strasbourg, France; 6 HydroSciences Montpellier, IRD, CNRS, Montpellier University, Hospital Hygiene and Infection Control Department, University Hospital of Montpellier, Montpellier, France

**Dear Editor,**

We read with interest the article by H. Ahmed and L.T. Joshi about the tolerance of *Clostridioides difficile* (CD) spores to disinfection with sodium hypochlorite disinfectant [1]. As reported in this study, the susceptibility of CD spores to chemical disinfectant is of paramount importance because the hospital environment is highly contaminated with *C. difficile* spores and can be a source of infection, especially in an outbreak setting [1]. The 2014 IDSA/APIC and 2018 ESCMID guidelines advocate for the use of hypochlorite or chlorine releasing agent for room disinfection of *C. difficile* infection (CDI) patients [2,3]. The tolerance of CD spores to hypochlorite raised unexpected concerns for infection control practitioners. Nevertheless, we identified some methodological shortcomings that weight the clinical implication of this study.

First, the choice of the strains was restricted to only two different PCR-ribotypes (027 and 012); the results of the experiments cannot be generalizable to the highly diverse population of CD [4].

Secondly, the method used to study the *in vitro* sporicidal activity raised some potential technical issues. The authors did not use a registered formulation of bleach-based disinfectant where stability was established, but an *in vitro* reconstituted solution using sterile water, where efficiency was presumed but not proven. In addition, they did not use a normalized procedure (e.g. the EN 17126) to prepare the spores' suspension and to measure the sporicidal activity [5]. The experiments did not include any control (e.g. a bacterial strain known to be susceptible to the 10 000 ppm sodium hypochlorite, and/or another disinfectant known to be active against all the tested CD spores) making difficult any relevant conclusion [6]. Indeed, the authors found that none of the tested hypochlorite concentration (i.e. 1000, 5000 or 10 000 ppm) displayed a significant sporicidal activity [6]. However, in another article recently published in *BMC Microbiology* (and co-signed by L.T. Joshi) and using the same CD strains (CD630 and R20291) and the same methodology, the authors found a reduction of at least 3 log of spore viability with hypochlorite 1 % after 10 min of contact time, which added in confusion about the true sporicidal activity of hypochlorite on CD [7].

Third, the last “real-life” section was an experimental approach, not sufficiently standardized to be theoretically relevant, and too distant from the reality to be clinically significant. The inoculum of 10^8^ spores used for the transfer to surgical scrubs and to patient gowns corresponded to a significant amount of spores that is not supported by any clinical claims. The absence of efficiency of the sodium hypochlorite on porous surfaces was expected in the study herein as *in vitro* experiments already showed non-significant reduction of CD spores. Furthermore, because of the lack of knowledge about the sodium hypochlorite putative action in this condition combining different factors [i.e. the penetration of hypochlorite in the personal protective equipment (PPE) and/or its interactions within the material], and given that such reusable PPE are expected to be laundered (with 60 °C – washing, drying and high-temperature ironing), the transposition of this experiment to the real-life hospital conditions appeared irrelevant.

The abnormal behaviour of some specific strains and spores is a relevant subject that must be carefully monitored. However, the transition from the bench to the hospital reality is quite difficult, especially when deviating from current standards. To date, the use of 1 : 10 dilution of sodium hypochlorite must be considered as a low-cost method whom the efficiency was well-recognized and -proven to disinfect the environment around CDI cases [4,8].

## Abbreviations

APIC: Association for Professionals in Infection Control and Epidemiology
CD: *Clostridioides difficile*
CDI: *Clostridioides difficile* infection
ESCMID: European Society of Clinical Microbiology and Infectious Diseases
IDSA: Infectious Diseases Society of America
PPE: personal protective equipment
